# Gene-based comparative analysis of tools for estimating copy number alterations using whole-exome sequencing data

**DOI:** 10.18632/oncotarget.15932

**Published:** 2017-03-06

**Authors:** Hyung-Yong Kim, Jin-Woo Choi, Jeong-Yeon Lee, Gu Kong

**Affiliations:** ^1^ Department of Pathology, College of Medicine, Hanyang University, Seoul, Republic of Korea; ^2^ Institute for Bioengineering and Biopharmaceutical Research (IBBR), Hanyang University, Seoul, Republic of Korea

**Keywords:** cancer CNV, CNA estimation, WES, NGS, copy number

## Abstract

Accurate detection of copy number alterations (CNAs) using next-generation sequencing technology is essential for the development and application of more precise medical treatments for human cancer. Here, we evaluated seven CNA estimation tools (ExomeCNV, CoNIFER, VarScan2, CODEX, ngCGH, saasCNV, and falcon) using whole-exome sequencing data from 419 breast cancer tumor-normal sample pairs from The Cancer Genome Atlas. Estimations generated using each tool were converted into gene-based copy numbers; concordance for gains and losses and the sensitivity and specificity of each tool were compared to validated copy numbers from a single nucleotide polymorphism reference array. The concordance and sensitivity of the tumor-normal pair methods for estimating CNAs (saasCNV, ExomeCNV, and VarScan2) were better than those of the tumor batch methods (CoNIFER and CODEX). SaasCNV had the highest gain and loss concordances (65.0%), sensitivity (69.4%), and specificity (89.1%) for estimating copy number gains or losses. These findings indicate that improved CNA detection algorithms are needed to more accurately interpret whole-exome sequencing results in human cancer.

## INTRODUCTION

The accumulation of genetic aberrations, ranging from the single nucleotide to the chromosome level, leads to various human diseases, including cancer. Several types of genetic aberrations, such as single nucleotide polymorphisms (SNP), insertions, deletions, duplications, and inversions, are associated with cancer. Copy-number alterations (CNAs) are defined as copy number variations (CNVs), including duplication, amplification, deletion, and homozygous deletion, in a specific genomic region in somatic cells [1]. Many CNAs have been identified in regions of the genome that contain multiple oncogenes and tumor suppressors [1–3], and these CNAs correlate with clinical outcomes and prognosis in various types of cancer, including colon, prostate, and breast cancers and leukemia [4–9]. These findings indicate that CNAs are important predictive and prognostic biomarkers in human cancer.

In recent years, high-throughput approaches, including array comparative genomic hybridization (aCGH) [10], SNP arrays [11, 12], and various forms of next-generation sequencing (NGS), such as whole-genome sequencing (WGS) and whole-exome sequencing (WES) [13–16], have been widely used to identify CNAs. NGS generates a great deal of information not only on genomic sequences and substitutional mutations, but also on CNVs or CNAs; this information can make possible the use of personalized medicine or precision oncology in treatment strategies. As the cost of WGS and WES decreases, they have become increasingly useful in cancer studies [17]. WES in particular allows for high coverage at a relatively low cost by targeting only the protein-coding regions of the genome, and may therefore be especially useful in clinical assays. However, the systematic noise associated with WES data, including signal variation caused by exon trapping bias, contamination by normal tissue, and multiple clones in the tumor sample, complicates the estimation of CNAs [18]. Additional bioinformatics tools are needed to more precisely estimate CNAs from WES data.

Several tools have been developed for estimating somatic CNAs using NGS data. For example, CNV-seq, ReadDepth, CNVnator, and HMMcopy use WGS data, and ExomeCNV, VarScan2, CoNVEX, CODEX, CoNIFER, and exome2cnv use WES data as inputs. Control_FREEC, saasCNV, ngCGH, and falcon use both WGS and WES [18, 19]. Each tool is characterized by distinct properties. For instance, ExomeCNV uses the log coverage ratio and requires a control set [20]. CoNIFER uses singular value decomposition methodology and does not require a control set [14]. VarScan2 uses pairwise comparison of read depth and population-based methods [21]. CODEX uses Poisson likelihood-based recursive segmentation and requires normalization using normal samples [22]. NgCGH computes a pseudo-CGH using simple coverage counting for the tumor relative to the normal sample. SaasCNV and falcon estimate allele-specific copy numbers based on B allele frequency [23, 24]. Studies have also been conducted to compare these CNA estimation algorithms [10, 25–27]. However, these studies compared WES-based CNAs with reference CNAs only at the exon level. Characterization of gene-based CNAs is very important in general cancer studies because knowledge of gene amplifications or deletions is crucial for diagnosis and treatment decisions.

In this study, we evaluated the ability of seven different CNA calling algorithms (ExomeCNV, CoNIFER, VarScan2, CODEX, ngCGH, saasCNV, and falcon) to estimate gene-based CNAs from WES data as compared to reference copy number data obtained from the SNP array (Genome-Wide Human SNP Array 6.0). Although CoNIFER was designed to identify CNVs at the population level, we used it here for comparison with tumor-normal pair CNA estimation. For these estimations, we used 419 breast cancer samples from The Cancer Genome Atlas (TCGA) project. The TCGA breast cancer dataset contains not only WES data, but also SNP6.0 array-based copy number data that were generated using the same DNA samples and which reflect the exact CNA status of each sample. By comparing the WES-based CNA estimations with the SNP6.0 copy numbers, we evaluated the accuracy and clinical applicability of the CNA estimation tools.

## RESULTS

### Estimated CNA segment sizes at the exon level differ among the algorithms

**Figure 1:**
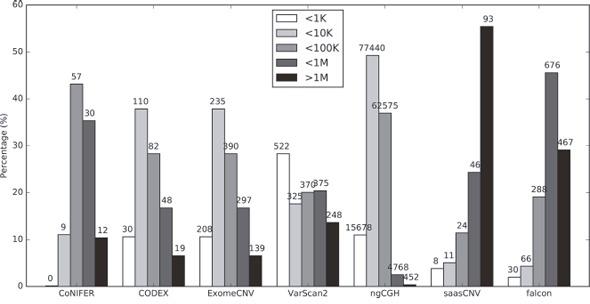
Distribution of CNA call sizes Sizes are stratified into 5 categories (<1K, 1K-10K, 10K-100K, 100K-1M, >1M). The total number of average CNA counts (gains and losses) for the 419 samples is displayed on the top of each bar. Neutral estimations are excluded from these numbers.

To evaluate the accuracy of conventional CNA detection tools at the gene level, we compared the accuracy of seven different WES-based CNA estimation algorithms (ExomeCNV, CoNIFER, VarScan2, CODEX, ngCGH, saasCNV, and falcon) to the SNP6.0 copy numbers generated using the same DNA samples from the TCGA dataset (Supplementary Figure 1). A summary of the 7 WES CNA estimation tools is presented in Table 1. We first assessed the CNA segment sizes obtained using these algorithms; each tool estimated a different distribution of CNA sizes (Figure 1, Supplementary Figure 2). Consistent with previous studies [26], CODEX and ExomeCNV estimated the CNAs evenly and with a normal distribution of sizes. In comparison, saasCNV and falcon estimated relatively longer CNAs, while ngCGH estimated shorter CNAs. These results reflect differences in the algorithms used in each tool. Because the average genomic size of a human gene is 10 ∼ 15 kbps and because many of the CNA segment sizes estimated based on WES were smaller, the results were merged to obtain representative copy numbers for one gene. Unexpectedly, the average CNA segment sizes per gene, which varied depending on the algorithm used, were large (ExomeCNV, 2.30 segments per gene; CoNIFER, 2.25; VarScan2, 2.28; CODEX, 2.10, ngCGH, 25.71; saasCNV, 2.17; and falcon, 2.33). Each tool incorporated specific numerical values into the estimation; these values were converted into copy numbers for each segment. Among the 7 estimation tools, ngCGH estimated the largest number of CNAs and, accordingly, the smallest CNA segment size (Figure 1, Supplementary Figure 2). These data indicate that CNA estimations based on a single WES BAM file differ depending on which estimation tool is used.

**Table 1:** Summary of the WES CNA estimation tools

Tool	OS	Language	Control required	Input format	Output format	Methodology characteristics	References
**CODEX**	Linux, Mac OS	R	No	BAM	Tab-delimited	Poisson log-likelihood ratio	Jiang Y and Zhang NR (2015) [22]
**CoNIFER**	Linux, Mac OS	Python	No	BAM/RPKM	Tab-delimited	SVD	Krumm *et al*. (2012) [14]
**ExomeCNV**	Linux, Mac OS, Windows	R	Yes	BAM/pileup/GTF	TXT, PNG	Log coverage ratio, CBS	Sathirapongsasuti *et al*. (2011) [20]
**VarScan2**	Linux, Mac OS, Windows	Java	Yes	BAM/pileup	Tab-delimited	CMDS, CBS	Koboldt *et al*. (2012) [21]
**ngCGH**	Linux,Mac OS,Windows	Python	Yes	BAM	Tab-delimited	Pseudo-CGH	
**saasCNV**	Linux,Mac OS,Windows	R	Yes	VCF	Tab-delimited	Allele specific by RD, BAF	Zhongyang *et al*. (2015) [23]
**falcon**	Linux,Mac OS,Windows	R	Yes	VCF	Tab-delimited	Allele specific by bivariate mixed Binomial	Chen *et al*. (2015) [24]

Detailed information regarding the specifications, algorithm, and reference for each tool is shown. BAM: Binary sequence alignment map, RPKM: Reads per kilobase per million mapped reads, GTF: General transfer format, SVD: Singular vector decomposition, CBS: Circular binary segmentation, CMDS: Correlation matrix diagonal segmentation, RD: Read depth, BAF: B allele frequency, CGH: Comparative genomic hybridization, VCF: Variant calling format.

### Gene-based CNA counts vary between the different algorithms and the reference

**Figure 2:**
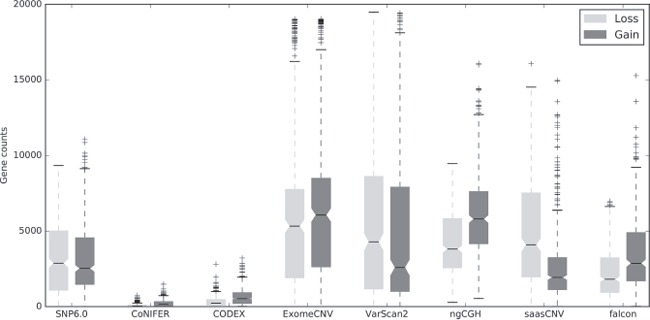
Numbers of gene-based copy number gains and losses generated by the WES CNA estimation tools compared to SNP6.0 Boxplots show the total numbers of gene-based copy number gains and losses estimated using the indicated algorithms. Gains are dark and losses are gray. Amplifications were merged with gains, and homozygous deletions were merged with losses.

We next converted each CNA estimation into gene-based copy numbers and compared the numbers of CNA gains and losses at the gene level for all 419 samples to the SNP6.0 copy numbers as a reference (Figure 2, Supplementary Figure 3). Each CNA estimation for the segments in a single gene was converted into a copy number, and total gain (amplification, +2; duplication, +1) and loss (deletion, -1; homozygous deletion, -2) counts were assessed. According to the SNP6.0 CNA gain and loss count distribution, the median CNA counts were approximately 2,500, suggesting that there were copy number losses or gains in approximately 5,000 genes in each breast cancer genome (Figure 2). VarScan2, ngCGH, and ExomeCNV estimated more CNAs compared to the reference, while CODEX and CoNIFER estimated fewer CNAs compared to the reference. Falcon estimated a similar number of CNAs compared to the reference (Figure 2). The gain and loss ratio was approximately 50% in the reference and in ExomeCNV. In contrast, CODEX, CoNIFER, ngCGH, and falcon had more gains than losses, whereas VarScan2 and SaasCNV had more losses than gains (Supplementary Figure 4). Overall, the tumor-normal pair estimation algorithms (VarScan2, ExomeCNV, ngCGH, saasCNV, and falcon) called more gains and losses than the tumor batch estimation algorithms (CODEX, CoNIFER) (Figure 2).

### Gene-based concordance between the reference and WES CNAs

**Figure 3:**
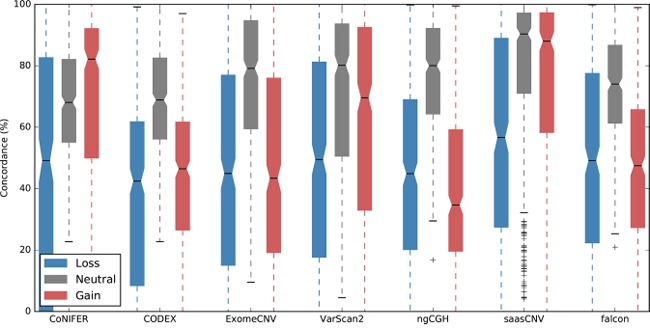
Overlap percentages for the WES-based CNAs and the reference CNA set The CNA estimations obtained using the seven tools were converted into gene based copy numbers (loss (−2, -1), neutral (0), or gain (1, 2)) and concordance with the SNP6.0 copy number reference was assessed.

To determine how many of the estimations obtained from each tool were consistent with the SNP6.0 copy numbers in this dataset, we compared the number of gene-based copy number gains or losses estimated by ExomeCNV, CoNIFER, VarScan2, CODEX, ngCGH, saasCNV, and falcon to the SNP6.0 copy numbers as a reference. There were an average of 1.2 SNP6.0 probe overlaps on each WES exon, implying that estimations of gene copy numbers in CNAs were similar between SNP6.0 and WES targets (Supplementary Table 1). The CNA algorithms differed from each other in this regard (Figure 3 and Supplementary Table 2). The tumor-normal pair estimation algorithms (VarScan2, ExomeCNV, and saasCNV) showed more than 50% gain and loss concordance, whereas the tumor batch estimation algorithm (CODEX) exhibited lower concordance with SNP6.0 (Figure 3). In contrast, the copy number neutral estimations produced by all algorithms showed similar concordance with the reference, and saasCNV was the most concordant with the SNP6.0 results (gain, 74.8% concordance; neutral, 78.3%; loss, 55.4%). Taken together, these results indicate that most gain and loss CNA estimations had large variations in concordance with SNP6.0 results, and concordance was better for gain estimation than for loss estimation. We also evaluated the ability of the CNA calling tools (CODEX, ngCGH, and falcon) that subcategorize gain and loss events (amplification, +2; duplication, +1; deletion, -1; homozygous deletion, -2) (Supplementary Figure 3) to estimate amplifications and homozygous deletions. However, their rate of concordance with the reference in estimating amplification was very low (Supplementary Figure 5), indicating that improvements are needed in CNA calling algorithms before such estimations can be made based on WES data.

### Concordance across CNA-calling algorithms

**Figure 4:**
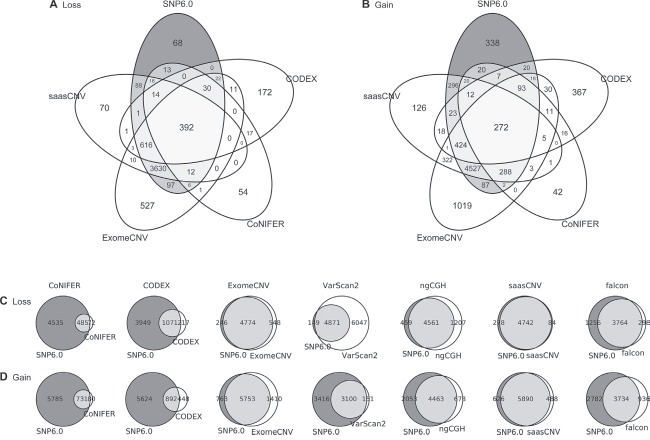
Gene-based Venn diagram The overlapping genes were calculated in the TCGA.AN.A0FL.01 sample. The Venn diagrams show the overlaps in **(A)** losses and **(B)** gains at the gene-level between the four selected CNV calling tools. Overlap between SNP6.0 copy numbers and each of the seven tools for **(C)** losses and **(D)** gains.

To investigate how many algorithms produced common estimations, we used a special case (TCGA.AN.A0FL.01) to construct a Venn diagram for the algorithms and the reference. Two representative tumor-normal pair methods (saasCNV and ExomeCNV) and two tumor batch methods (CoNIFER and CODEX) were used for this analysis (Figure 4A, 4B, Supplementary Figure 6). SaasCNV and ExomeCNV generated a larger number of correct common estimations compared to CODEX and CoNIFER, but the tumor-normal pair methods also generated many wrong estimations (Figure 4A, 4B, Supplementary Figure 6). We also compared the common estimations produced by each of the 7 algorithms to those in the reference using a Venn diagram (Figure 4C, 4D). Again, saasCNV and ExomeCNV were better at estimating gains and losses than the other tools. The concordance of tumor batch methods (CODEX and CoNIFER) was relatively low; these programs generated a small number of correct estimations and many wrong estimations. Analysis of the shared estimations for all 419 cases between the reference and the 7 algorithms revealed that SaasCNV was the most similar to the SNP6.0 reference (Table 2).

**Table 2:** Similarity between SNP6.0 CNA and the estimation algorithms

	SNP6.0	saasCNV	VarScan2	ExomeCNV	ngCGH	falcon	CoNIFER	CODEX
**SNP6.0**	1.000	0.634	0.565	0.464	0.506	0.305	0.155	0.148
**saasCNV**		1.000	0.514	0.475	0.506	0.320	0.100	0.100
**VarScan2**			1.000	0.457	0.473	0.268	0.101	0.099
**ExomeCNV**				1.000	0.441	0.215	0.072	0.080
**ngCGH**					1.000	0.418	0.190	0.133
**falcon**						1.000	0.152	0.113
**CoNIFER**							1.000	0.247
**CODEX**								1.000

Average Pearson correlations of the estimation of CNAs in 419 TCGA breast cancer samples. (19,780 gene copy numbers state: amplification, +2; duplication, +1; neutral, 0; deletion, -1; homozygous deletion, -2).

**Figure 5:**
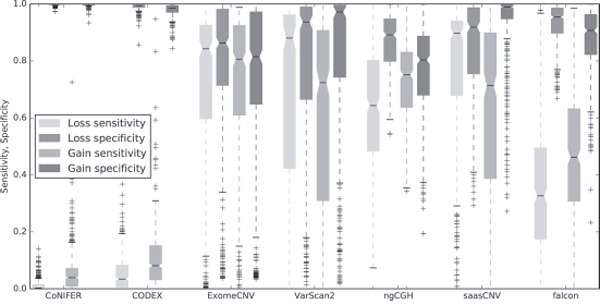
Sensitivity and specificity of CNA loss and gain estimations Boxplots of the sensitivity and specificity for losses and gains are shown for each tool and compared to the SNP6.0 results.

Next, we calculated the sensitivity and specificity of each algorithm using the CNA estimations for all samples. To do this, we assessed whether each estimation was a true positive, true negative, false positive, or false negative using the 19,780 genes for all 419 samples available in SNP6.0. As shown in the boxplot in Figure 5, sensitivity and specificity percentages for saasCNV, ExomeCNV, and VarScan2 were higher than approximately 50%, whereas CODEX and CoNIFER had lower sensitivities. The high specificity of CODEX and CoNIFER reflected the small number of gain or loss estimations they generated. Falcon also showed low sensitivity and high specificity, and the loss sensitivity of saasCNV was much higher than that of the other tools.

## DISCUSSION

In the present study, we evaluated 7 CNA estimation tools converted at the gene level using WES data for 419 paired breast cancer tumor and normal tissue sample pairs from TCGA to SNP6.0 copy numbers as a reference. SaasCNV, ExomeCNV, and VarScan2 showed better concordance with the reference and had higher sensitivity and specificity in estimating gains and losses than the other tools. The number of estimations produced by the different algorithms varied: VarScan2 and ExomeCNV tended to overestimate, while CODEX and CoNIFER tended to underestimate, compared to the reference. Furthermore, saasCNV had the highest concordance with the SNP6.0 results among the algorithms tested. These results characterize the properties and demonstrate the limitations of each tool for estimating CNAs using WES data.

Using SNP6.0 copy number as the gold standard for CNA estimation, our findings suggest that tumor-normal pair methods should be used to estimate CNAs based on WES data; CNA estimations generated with the tumor-normal pair methods saasCNV, ExomeCNV, and VarScan2 had higher concordance and sensitivity compared to the reference than those generated by the tumor batch methods CODEX and CoNIFER. Our data also suggest that saasCNV, an allele-specific CNA estimation tool, may be more valuable than other algorithms for estimating gains and losses in copy numbers using WES data. A major advantage of the tools examined here is their ability to estimate copy-neutral LOH (loss of heterozygosity), but we could not examine this function relative to the reference here because SNP6.0 was not used for LOH estimation in the TCGA dataset. None of WES-based CNA calling algorithms tested were able to accurately estimate the amplification and homozygous deletion subcategories of gains and losses compared to the reference, and a CNA calling algorithm that can make those predictions accurately is needed for cancer studies. In addition, the concordance of the current gain and loss CNA estimation tools appears to vary widely, and additional studies may be needed to identify the sample data characteristics that contribute to high concordance.

Our results here both share similarities with and differ from the findings of previous reports that have evaluated CNA detection tools. Nam *et al*. previously examined ExomeCNV and VarScan2 using WES data from 150 patients with SNP arrays and WGS as reference methods [25]. They found that ExomeCNV was less than 40% concordant for gains and less than 50% concordant for losses, while concordance for VarScan2 was approximately 50% for both gains and losses. We obtained similar results here for our gene-based CNA estimation (ExomeCNV, 47.1% concordance in gains, 46.4% in losses; VarScan2, 61% in gain, 49.2% loss). Tan *et al*. also evaluated the concordance of CNA estimation tools using WES data for 33 samples with WGS as a reference and found that the concordance of CoNIFER was very low (3.18%) [26]. In contrast, we found that CoNIFER was moderately concordant (67.5% in gains, 46.2% in losses), but also had high specificity and low sensitivity. These differences between studies may be due to the differences in the gene-based CNA estimation methods used here compared to those used in previous studies.

Notably, CNAs were estimated at the gene level in our study, while most previous CNA estimation tool comparison studies performed estimations at the exon level. Because a single gene can have many exons, it is difficult to accurately identify copy number changes in specific genes, and estimation tools can return many different copy numbers for a single gene. Gene level CNA estimation methods may therefore be particularly helpful in determining whether a specific oncogene is amplified in the clinical setting. In this study, we used the rounded arithmetic mean of the copy numbers for each segment on single genes generated using the various methods to characterize CNAs for each gene. Furthermore, compared to previous studies, we used more WES samples (419 tumor-normal pairs) and gene level estimations for more convenient clinical interpretation. We also confirmed the efficacy of the current CNA estimation tools for diagnosis by assessing their sensitivity and specificity. Although tumor-normal pair estimations, particularly saasCNV, were more reliable than tumor batch estimations, their sensitivity and specificity were lower than desired; our present results should therefore be interpreted with caution.

Collectively, our findings indicate that current WES CNA detection algorithms should be applied more carefully depending on the specific aims of different studies, and additional improvements and tools are needed to improve the accuracy of WES CNA detection.

## MATERIALS AND METHODS

### Datasets analyzed

We extracted 419 of the 471 breast cancer patient samples in The Cancer Genome Atlas (TCGA) for use in this study based on QC and SNP array (Genome-Wide Human SNP array 6.0) copy number data availability [3]. WES BAM files were acquired from the Cancer Genomics Hub (CGHub) through NCBI dbGaP authorized access. Each BAM file was mapped to GRCh37-lite for human genome reference. Two exon capture kits were used in this dataset: Agilent (120 pairs) and Nimblegen (299 pairs). Each CNA estimation tool used the information in these kits (BED file) for WES CNA estimation.

“SNP and copy number level 3 GISTIC” data, which provided thresholded copy number information for each gene, including amplification (+2), duplication (+1), neutral (0), deletion (−1), and homozygous deletion (−2), were also provided for the same patients in the TCGA dataset. This information was used as the gold standard for the evaluation of each estimation tool.

### Execution of CNA estimation tools

The CODEX, CoNIFER, ExomeCNV, VarScan2, ngCGH, saasCNV, and falcon CNA estimation tools were selected for evaluation in this study. A summary of these WES CNV estimation tools is provided in Table 1. CODEX and CoNIFER used WES BAM files and the kit information BED file as inputs; these programs did not require normal data. CODEX used normal data for normalization. ExomeCNV used kit information BED files and coverage files as inputs. Coverage files were generated using the GATK DepthOfCoverage program with normal and tumor WES BAM files [28]. VarScan2 used pileup files, which were generated using normal and tumor WES BAM files in SAMtools [29], as inputs. Each estimation used default threshold settings. NgCGH used normal-tumor pair BAM files directly. SaasCNV and falcon required VCF files, which were generated using VarScan2, for allele-specific CNA estimations. Details regarding versions, processes, and options are described in the Supplementary Materials and Methods.

### Gene-based evaluation

Each tool estimated CNAs by genomic region. We subsequently annotated the results at the exon level using BEDTools [30]. For gene-based evaluation, the copy numbers for each exon were merged to generate one gene copy number based on the rounded average of the copy number values. For example, if the CNAs of gene *DDX11L1* exons were [−1, 1, 0, 1, 0, 1, 1, 1, 1, 1], the average was 0.6, and the rounded average “1” was used as the copy number for this gene. We compared the CNA values obtained for each gene using the selected estimation tools to the SNP6.0 copy number for all 419 patients. The concordance of each tool with the reference was statistically analyzed using *t*-tests in R statistical software; *P* values less than 0.05 were considered statistically significant.

## SUPPLEMENTARY MATERIALS FIGURES AND TABLES


