# Supplementary material for: Gene-based comparative analysis of tools for estimating copy number alterations using whole-exome sequencing data
Source: Oncotarget. 2017 Mar 6;8(16):27277–85. doi: 10.18632/oncotarget.15932 (PMC5432334; doi:10.18632/oncotarget.15932)
Supplement: Supplementary file 1 [file oncotarget-08-27277-s001.pdf]

Gene-based comparative analysis of tools for estimating copy number alterations using whole-exome sequencing data

SUPPLEMENTARY MATERIALS

SUPPLEMENTARY DATA

Procedural, version, and options information

CoNIFER

Version 0.2.2 was used. RPKM files were generated for each tumor BAM using the "analyze" command with Agilent/Nimblegen BED file and the "--svd 6" option. The "call" command was used to generate output text files for each sample. We used the "state" column for CNA estimation. Gene IDs for each CNA region were generated using the BEDtools "intersect" command with UCSC Table Browser's gene export. We used the "mygene" Python package to convert RefSeq ID to NCBI Gene ID.

CODEX

Version 1.0 was used (R 3.2.0). Optimal K values were generated for each sample using normal BAM files. Final call result text files were generated using the "segment" function with the optimal K value, tumor BAM files, and the "lmax=200" setting. We used the "cnv" column for CNA estimation. Gene IDs were generated as described for CoNIFER.

ExomeCNV

Version 1.4 was used (R 3.2.0). Normal and tumor coverage were generated for each sample using the GATK "DepthOfCoverage" command with Human reference fasta (GRCh-37), BAM, and Agilent/Nimblgen BED files. CNA results were generated for each sample using the "classify.eCNV" function with the normal/tumor coverage option selected and the following settings: min.spec=0.9999, min.sens=0.9999, option='spec', c=0.5, l=70. We then combined these results into larger segments using DNAcopy's "multi.CNV.analyze" function with the following settings: coverage.cutoff=5, min.spec=0.99, min.sens=0.99, option='auc', c=0.5. Copy numbers from the ".segment.copynumber.txt" file were used for CNA estimation. Gene IDs were generated as described for CoNIFER.

VarScan2

Version 2.2.3 was used (Java 1.8.0). Normal and tumor pileup files were generated for each sample using the SAMtools "pileup" command with Human reference fasta (GRCh-37) and BAM files. Text files with copy number results were generated using the "copynumber" command with the normal/tumor pileup file and the "copyCaller" command. These results were then combined into larger segments using DNAcopy's smooth and segment function. After re-centering and merging adjacent segments using "mergeSegments.pl", we used the "event type" column for CNA estimation. Gene IDs were generated as described for CoNIFER.

ngCGH

Version 0.4.4 was used (Python 2.7.10). CNA estimations were generated for each sample using tumor and normal BAM files and default options. Because this method calls log2 ratio rather than CNAs, rounded average log2 ratios for each gene region were used for CNA evaluation with a correction for tumor purity (TCGA breast cancer average tumor purity is 60%). Gene IDs were generated as described for CoNIFER.

saasCNV

Version 0.3.4 was used (R 3.2.0). Normal and tumor pileup files were generated for each sample using the SAMtools "pileup" command with Human reference fasta (GRCh-37) and BAM files. VCF files were generated using VarScan2's "somatic" command with tumor/normal pileup files and the "--output-vcf 1" option. Copy number results were generated using saasCNV's "cna.call" function with VCF files and the following settings: maxL=2000, N=1000, pvalue.cutoff=0.05. We used the "CNV" column for CNA evaluation. We did not include LOH to loss. Gene IDs were generated as described for CoNIFER.

Falcon

Version 0.2 was used (R 3.2.0). Tumor/normal VCF files were generated for each sample using the method described for saasCNV. Copy number results were generated using falcon's "getASCN' function with VCF files and default options. The "cns1" and "cns2" values returned refer to allele-specific copy numbers. We used the rounded average of "(cns1 + cns2)" in each gene region for gene-based CNA evaluation; "2" is neutral, "3" and above are total gains, and "1" and below are total losses. Gene IDs were generated as described for CoNIFER.

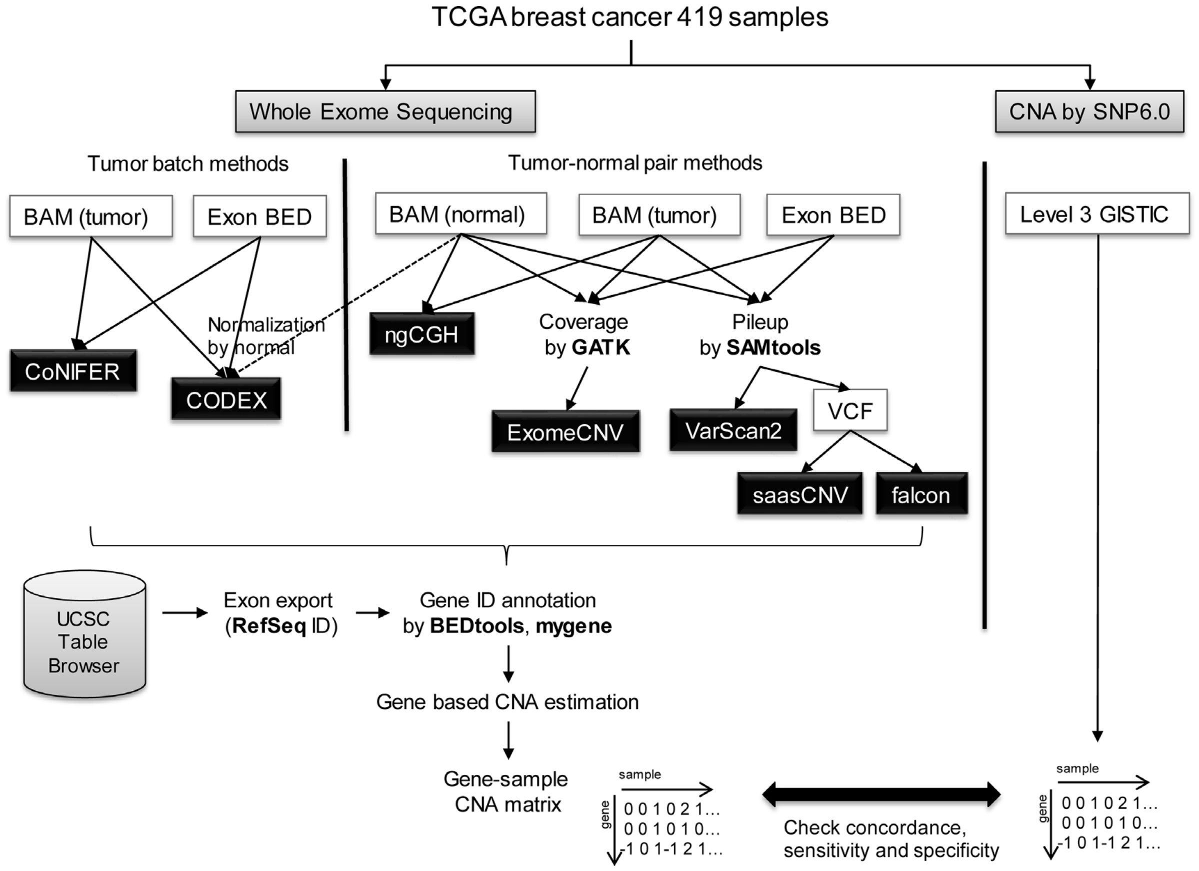

Supplementary Figure 1: Diagram of the overall CNA estimation process for the seven tools. Whole exome sequencing (WES) and SNP6.0 data were obtained from TCGA breast cancer dataset. CNAs were estimated based on WES data using the indicated tools; the concordance, sensitivity, and specificity of these tools compared to the SNP6.0 results were then analyzed.

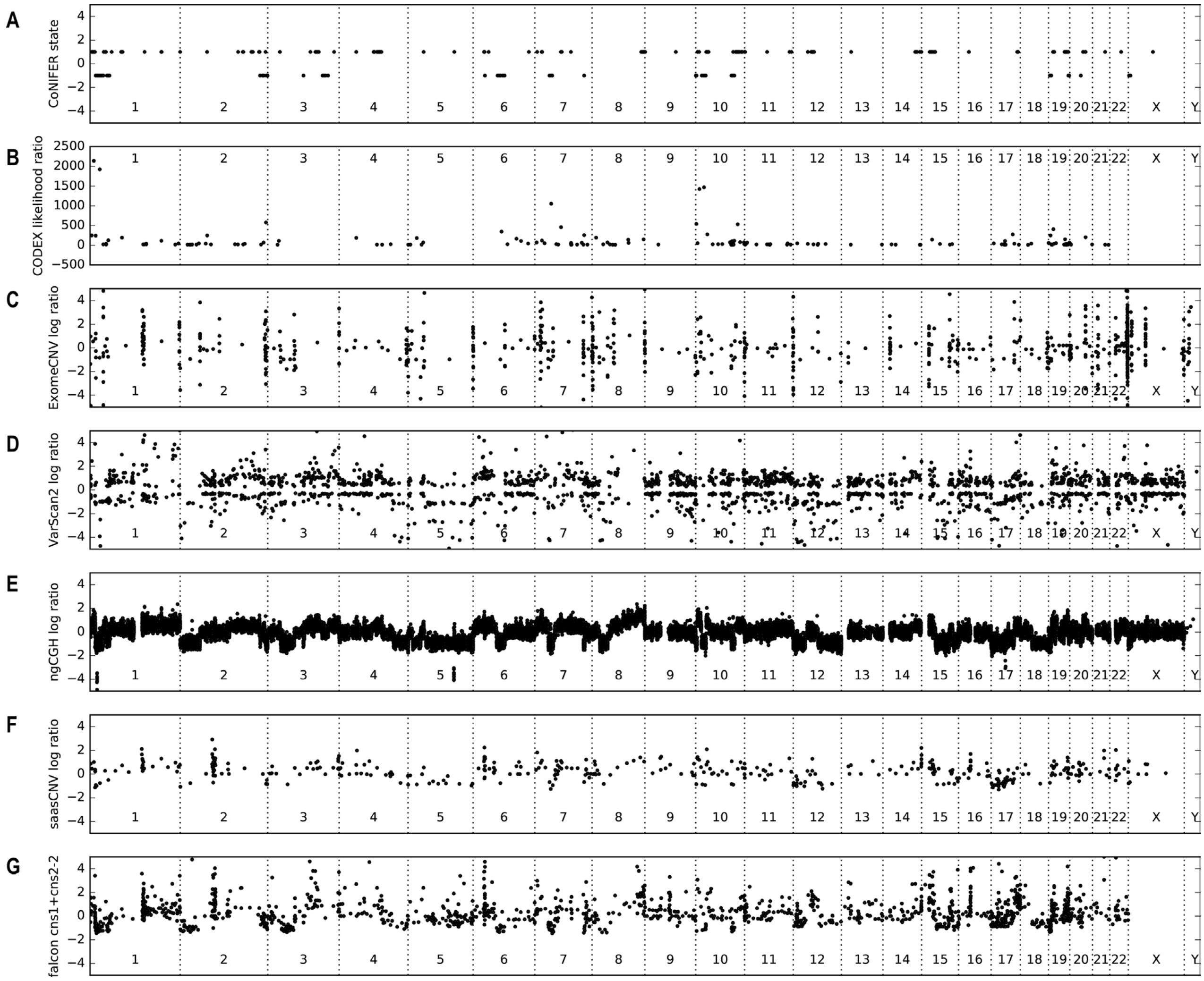

Supplementary Figure 2: Chromosome CNA estimations. **(A)** CNA status estimated by CoNIFER. **(B)** Likelihood ratio of CNA segment generated by CODEX. Log ratio of CNA segments generated by **(C)** ExomeCNV, **(D)** VarScan2, and **(E)** ngCGH. **(F)** Adjusted mean log ratio generated by saasCNV. **(G)** cns1+cns2 generated by falcon (sample ID: TCGA-A2-A0CT).

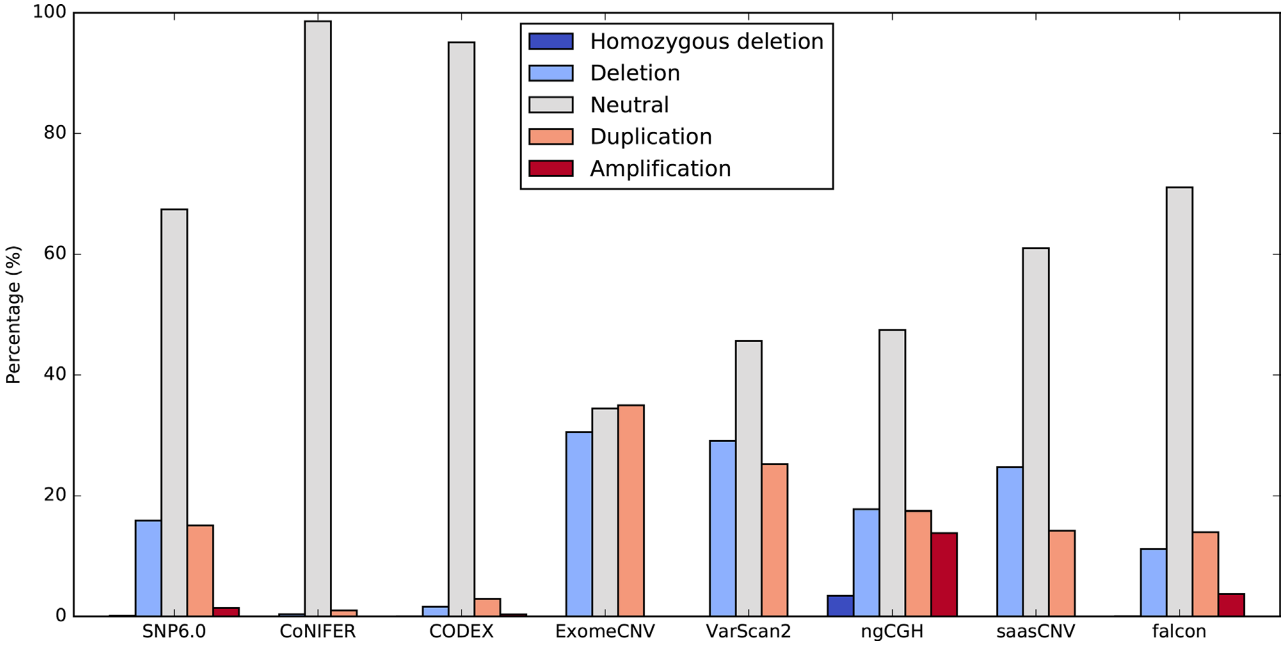

Supplementary Figure 3: Gene-based CNAs identified using each method. Percentages of the indicated types of CNAs estimated by each tool are shown in the bar graph. Only CODEX, ngCGH, and falcon generated estimations of amplifications and homozygous deletions.

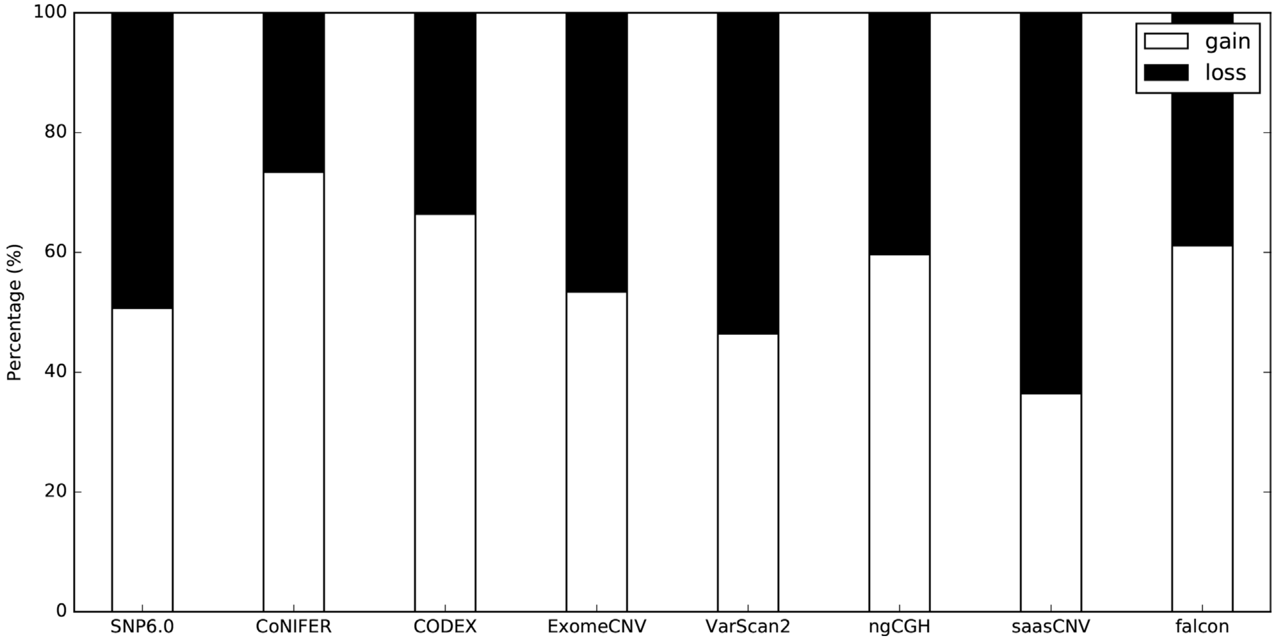

Supplementary Figure 4: Ratios of gene-based gain and loss CNAs at the genomic scale identified using each method. Gains are in white and losses in black. Amplifications were merged with gains, and homozygous deletions were merged with losses.

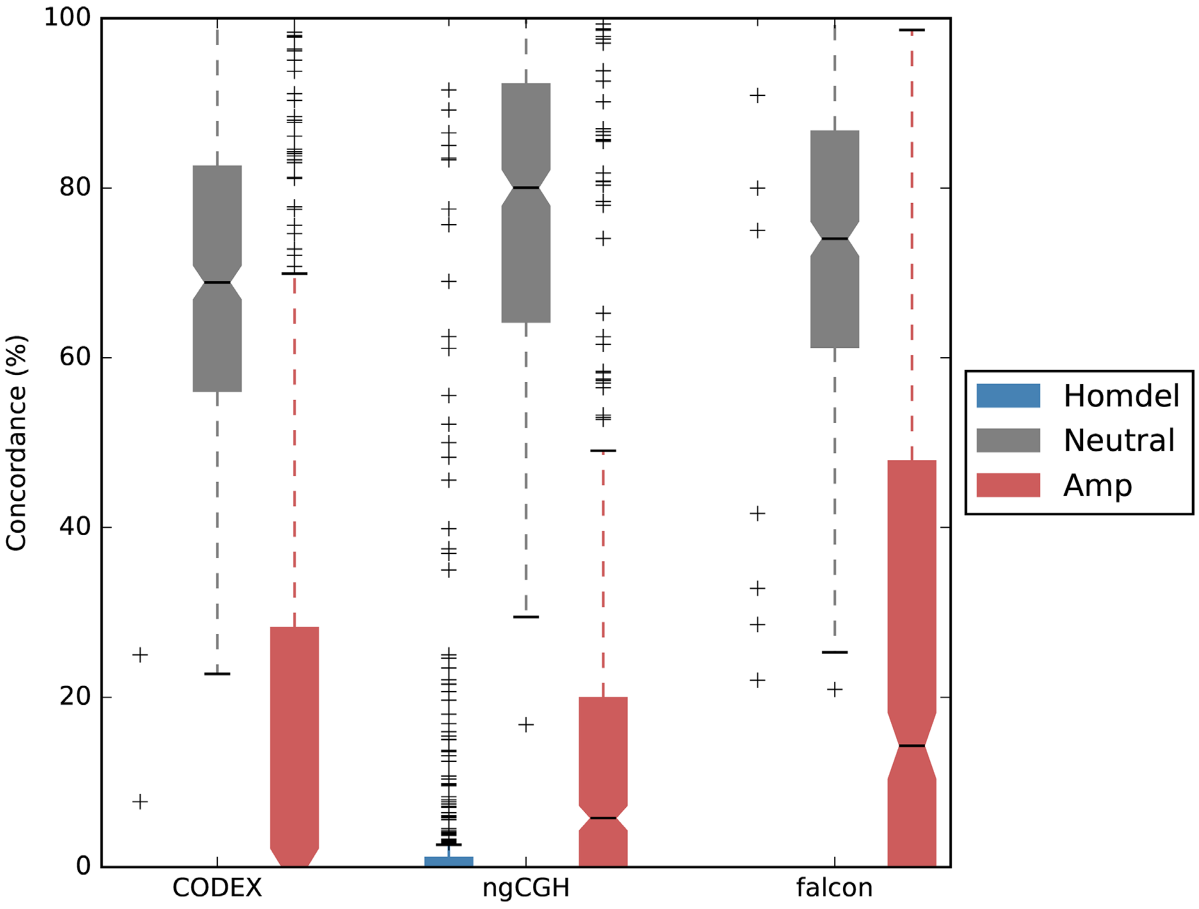

Supplementary Figure 5: Overlap percentages for amplification and homozygous deletion with the reference CNA set. The concordance of the estimations for the amplification and homozygous deletion CNA subcategories generated by CODEX, ngCGH, and falcon with neutral estimation are shown.

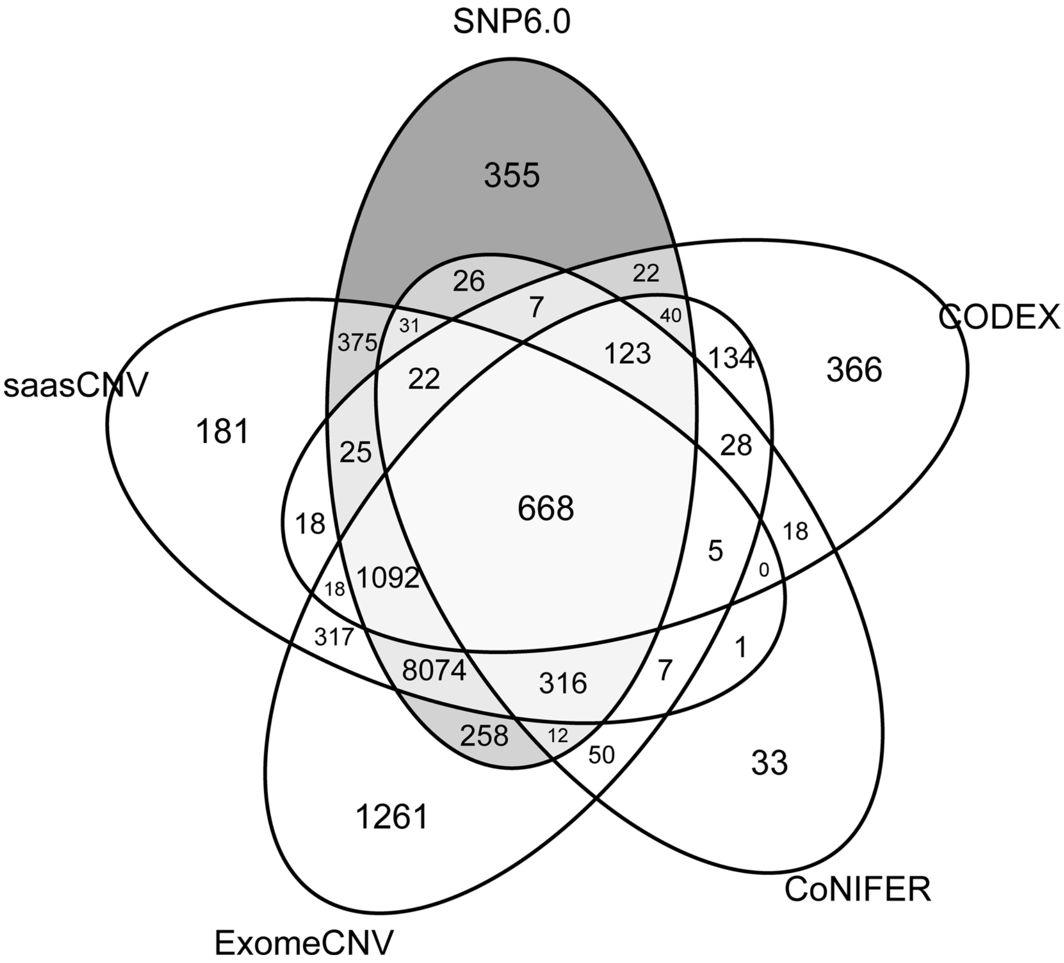

Supplementary Figure 6: Overlap in CNAs at the gene level among the four CNA estimation tools. The concordance of all CNAs (gain, neutral, loss) in a single TCGA.AN.A0FL.01 sample is shown in a Venn Diagram.

Supplementary Table 1: Overlap between SNP6.0 probes and WES whole-exon targets

Probe	Counts	Overlap counts	Average counts of SNP probes in overlapped exon
SNP6.0 probe	1,878,340		
Agilent WES exon	195,905	22,438 (11.4%)	1.229
Nimblegen WES exon	194,885	19,918 (10.2%)	1.204

Total numbers of SNP6.0 probes and whole-exome targets from Agilent or Nimblegen WES, as well as numbers of overlapping targets, are shown. The average numbers of SNP probes in the overlapping exon were calculated based on the overlap counts between the SNP6.0 probes and WES whole-exome targets.

Supplementary Table 2: Significance of overlap between WES-based CNAs and the reference CNA set

		t-test *P*-value		
		**Loss**	**Neutral**	**Gain**
**CODEX**	**ngCGH**	0.0042	< 2.9e-11	0.0230
**CODEX**	**saasCNV**	< 5.4e-12	< 1.6e-09	< 4.2e-53
**CODEX**	**VarScan2**	< 1.6e-05	0.2238	< 7.6e-17
**CODEX**	**CoNIFER**	0.0078	0.7026	< 2.1e-29
**CODEX**	**falcon**	< 1.7e-05	0.0030	0.411
**CODEX**	**ExomeCNV**	0.0023	< 5.4e-05	0.1104
**ngCGH**	**saasCNV**	< 1.7e-05	0.4018	< 8.5e-61
**ngCGH**	**VarScan2**	0.1062	< 4.6e-05	< 9.4e-23
**ngCGH**	**CoNIFER**	0.8024	< 2.3e-12	< 2.8e-36
**ngCGH**	**falcon**	0.1098	0.0004	< 6.0e-05
**ngCGH**	**ExomeCNV**	0.7182	0.0613	0.0007
**saasCNV**	**VarScan2**	0.0097	< 3.8e-05	< 6.3e-10
**saasCNV**	**CoNIFER**	0.0006	< 2.6e-10	0.0009
**saasCNV**	**falcon**	0.0095	0.0004	< 3.6e-39
**saasCNV**	**ExomeCNV**	0.0002	0.0223	< 1.6e-34
**VarScan2**	**CoNIFER**	0.2563	0.1292	0.0054
**VarScan2**	**falcon**	0.9862	0.2517	< 3.0e-10
**VarScan2**	**ExomeCNV**	0.2367	0.0265	< 1.1e-09
**CoNIFER**	**falcon**	0.2669	0.0009	< 5.8e-20
**CoNIFER**	**ExomeCNV**	0.9445	< 1.2e-05	< 1.8e-18
**falcon**	**ExomeCNV**	0.2459	0.1892	0.8290

The statistical significance of differences in concordances between CNA estimations for each tool with SNP6.0 results was analyzed using *t*-tests.
